# Icon arrays reduce concern over COVID-19 vaccine side effects: a randomized control study

**DOI:** 10.1186/s41235-022-00387-5

**Published:** 2022-05-07

**Authors:** Madison Fansher, Tyler J. Adkins, Poortata Lalwani, Aysecan Boduroglu, Madison Carlson, Madelyn Quirk, Richard L. Lewis, Priti Shah, Han Zhang, John Jonides

**Affiliations:** 1 Department of Psychology, University of Michigan, 530 Church St, East Hall, Room 1004i, Ann Arbor, MI 48109 USA; 2 Department of Psychology, Bogazici University, Istanbul, Turkey; 3 Department of Linguistics, University of Michigan, Ann Arbor, MI 48109 USA; 4 Weinberg Institute for Cognitive Science, University of Michigan, Ann Arbor, MI 48109 USA

## Abstract

On April 13, 2021, the CDC announced that the administration of Johnson and Johnson’s COVID-19 vaccine would be paused due to a rare blood clotting side effect in ~ 0.0001% of people given the vaccine. Most people who are hesitant to get a COVID-19 vaccine list potential side effects as their main concern (PEW, 2021); thus, it is likely that this announcement increased vaccine hesitancy among the American public. Two days after the CDC’s announcement, we administered a survey to a group of 2,046 Americans to assess their changes in attitudes toward COVID-19 vaccines. The aim of this study was to investigate whether viewing icon arrays of side effect risk would prevent increases in COVID-19 vaccine hesitancy due to the announcement. We found that using icon arrays to illustrate the small chance of experiencing the blood clotting side effect significantly prevented increases in aversion toward the Johnson and Johnson vaccine as well as all other COVID-19 vaccines.

**Supplementary Information:**

The online version contains supplementary material available at 10.1186/s41235-022-00387-5.

## Significance statement

Vaccination is one of the most effective ways to reduce the risk of serious illness due to COVID-19. Given that many Americans are hesitant to receive a COVID-19 vaccine, and that a majority cite side-effect risk as their main concern, it is critical that we carefully communicate information about COVID-19 vaccine side effects to the public. Across two randomized control studies we find that viewing icon arrays illustrating the small risk of experiencing blood clots from the Johnson and Johnson vaccine decreased aversion toward COVID-19 vaccines. This suggests that icon arrays illustrating small risks should be used when communicating information about COVID-19 vaccine side effects.

## Introduction

On April 13, 2021, the CDC paused administration of Johnson and Johnson’s (J&J) COVID-19 vaccine to review six reports of a serious blood clotting condition out of the ~ 6.8 million doses that had been administered (CDC, 2021). People generally struggle to comprehend probabilistic risk information when it is depicted numerically (Peters, 2012; Slovic et al., 2000) and often overestimate the occurrence of consequential but unlikely events, including those associated with vaccination (Reyna, 2004). Such risks may evoke high dread when viewed by non-experts, socially amplifying small risks to society-level problems (e.g., Slovic & Weber, 2002). It is possible that the CDC’s announcement increased vaccine hesitancy due to these psychological biases (Slovic & Weber, 2010) especially considering that of those who are hesitant to be vaccinated for COVID-19, 72% cite concern over side effects as the main contributor (Funk & Tyson, 2021). Two days after the CDC’s announcement, we investigated how probability language and data visualizations incorporated into the announcement might have alleviated potential increases in aversion toward both the J&J and *all* COVID-19 vaccines.

**Fig. 1:**
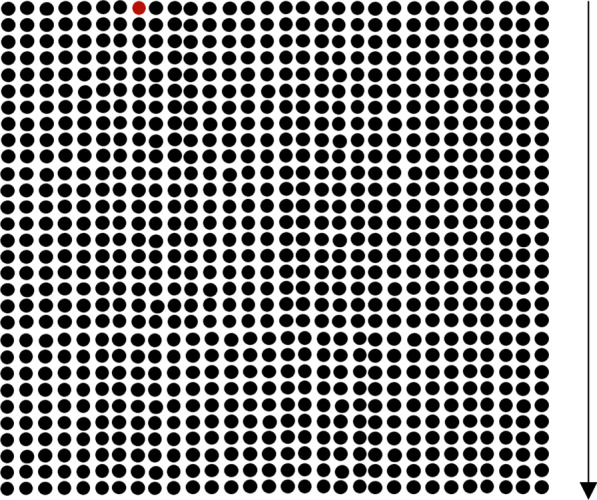
An icon array illustrating the 1 (red dot) in 900 chance of experiencing a side effect due to a treatment. The icon array in Experiment contained 1 million dots, one of them red, that participants had to scroll through if assigned to a visualization condition. The arrow on the right represents how participants had to scroll through the array of dots, but this arrow was not part of the original figure

People interpret risk differently depending on how it is presented (see Reyna & Brainerd, 2008 for a review). Thus, in Experiment 1 we examine the influence of language (i.e., expressing the probability as a ratio, percentage, or single number) on changes in vaccine aversion. We also tested whether viewing an icon array depicting the small risk of experiencing the blood-clotting side effect would prevent increases in vaccine aversion. Prior work suggests that understanding of risk may be improved with the use of such displays (Tait et al., 2010; Waters et al., 2007a, b; see Fig. 1). Graphical depictions of risk in the form of icon arrays are thought to be beneficial because they highlight both the numerator (the number of times X has happened) and denominator (the number of time X *could* have happened) (for a review, see Garcia-Retamero & Cokely, 2013). People often neglect the information presented in the denominator when interpreting risk information, thus overestimating the occurrence of risks (Garcia-Retamero & Galesic, 2009; Reyna, 2004). Icon arrays have been shown to be especially helpful with communicating risks to people with low numeracy (see Galesic et al., 2009). The effectiveness of icon arrays is usually tested in hypothetical scenarios in which participants compare treatment benefits and side effects (see Galesic et al., 2009; Garcia-Retamero & Galesic, 2010; Hawley et al., 2008). The literature on whether real-world and hypothetical decisions differ provides mixed evidence, usually in the context of risky decision making (Kühberger et al., 2002). One novel contribution of the current investigation is that we examine the influence of icon arrays on risk perception in a real-world context, which is particularly important because of the immediate public health implications of vaccination. Another unique contribution of this investigation is that we use icon arrays to illustrate a very small risk (~ 1 in 1 million). Typically, in prior investigations the focus has been on much higher side-effect risks. For example, Tait et al. (2010) discussed a 5% side effect risk.

In Experiment 2, we further explore how different types of icon arrays influence vaccine attitudes by adding a condition in which participants viewed the relative risk of experiencing side effects to lives saved by the vaccine. Across both studies, we found evidence that viewing icon arrays prevented increases in aversion to the J&J vaccine and possibly to *all* COVID-19 vaccines.

## Experiment 1

Experiment 1 examined how probability language would influence changes in aversion to the J&J and all COVID-19 vaccines. The experiment also examined whether the presence of an icon array illustrating side-effect risk would prevent increases in vaccine hesitancy (Fig. 1).

### Methods

#### Participants

Data were collected from 1143 participants from Amazon MTurk. Ninety participants were excluded from the analyses for inattentiveness, leaving 1052 participants. See demographics in Table 1.

**Table 1:** Demographic characteristics of participants in Experiment 1

Age M (SD)	Gender		Education	
38.81 (14.37)	Female	61.31%	Some High School	.48%
	Male	38.02%	High School	7.7%
	Other	.7%	Some College	12.07%
			2-years degree	9.31%
			4-years degree	55.22%
			Advanced degree	15.21%

#### Design and materials

Experiment 1 used a 3 (probability expression) by 2 (visualization presence) between-subjects design. Participants were randomly assigned to read the probability of incurring the J&J side effect as a percentage (0.0001% of people), ratio (6 in 6.8 million people), or single number (6 people). As an example, the following vignette was shown to those assigned to the single number condition:

The US Centers for Disease Control and Prevention and the US Food and Drug Administration are recommending that the USA pause the use of Johnson & Johnson's COVID-19 vaccine over six reported US cases of a "rare and severe" type of blood clot.

Participants were also assigned to view either an icon array depicting the risk of experiencing the blood clotting side effect, or no icon array. The icon array contained one million dots, one of which was red, representing the 0.0001% probability of experiencing the side effect reported by the CDC. The icon array had labels on the left side of the image, breaking up the visualization into of multiples of 100,000 (e.g., “100,000”, “200,000”, etc.). All of the dots were large enough that they were clearly visible to participants (see OSF for materials).

Participants read the following description:

In the chart presented below, we illustrate the proportion of people who experience the blood clotting side effect after getting the Johnson & Johnson vaccine. Each dot represents a single person who received the vaccine. One of these dots is red. The red dot represents a person who experiences the blood clotting side effect. Out of all the dots below, only one will experience the side effect.

#### Procedure

Participants provided informed consent, reported their vaccination status, and were shown one of three vignettes about the CDC’s new guidelines for the J&J vaccine (depending on condition). If assigned to the icon array condition, the participants viewed this information after reading the vignette. Participants then self-reported their change in attitudes toward the J&J and *all* COVID-19 vaccines with slider scales from 0 to 100, totaling 6 items:

1. This announcement would make me more hesitant to get (the J&J/any COVID-19) vaccine (shown only to vaccinated participants)
2. This announcement has made me more hesitant to get (the J&J/any COVID-19) vaccine (shown only to unvaccinated participants)
3. I'm more concerned about the safety of (the J&J/any COVID-19) vaccine after this announcement
4. Compared to yesterday, I'm less likely to recommend that my friends and family get (the J&J/any COVID-19) vaccine

Lastly, participants completed the subjective numeracy scale (Fagerlin et al., 2007), to be used as a covariate in the modeling of the data. Participants were compensated $1, and all procedures were determined to be exempt by the University of Michigan IRB. Readers may access our surveys, data, and code at https://osf.io/psvmw/?view_only=7a63dae90fb34411b49a9ffaa7e0d8e4.

#### Modeling methods

Slider scale responses to increases in vaccine hesitancy, safety concern, and reluctance to recommend vaccination items were rescaled from 0–100 to 0–1. These items were highly correlated (r > 0.8) and were averaged to create two composite changes in vaccine aversion scores, one for the J&J vaccine and one for *all* COVID-19 vaccines. It is reasonable to assume that the announcement may influence perceptions of the J&J vaccine; however, it is unknown whether the announcement would influence change in attitudes toward other vaccines that were not associated with the reported side effects. Thus, we modeled change in aversion to the J&J vaccine and all COVID-19 vaccines separately, even though they were moderately correlated (r = 0.48 in Exp. 1, r = 0.61 in Exp. 2). When interpreting the composite scores, 1 indicates a large increase in aversion and 0 indicates no increase in aversion toward the vaccine(s).

**Fig. 2:**
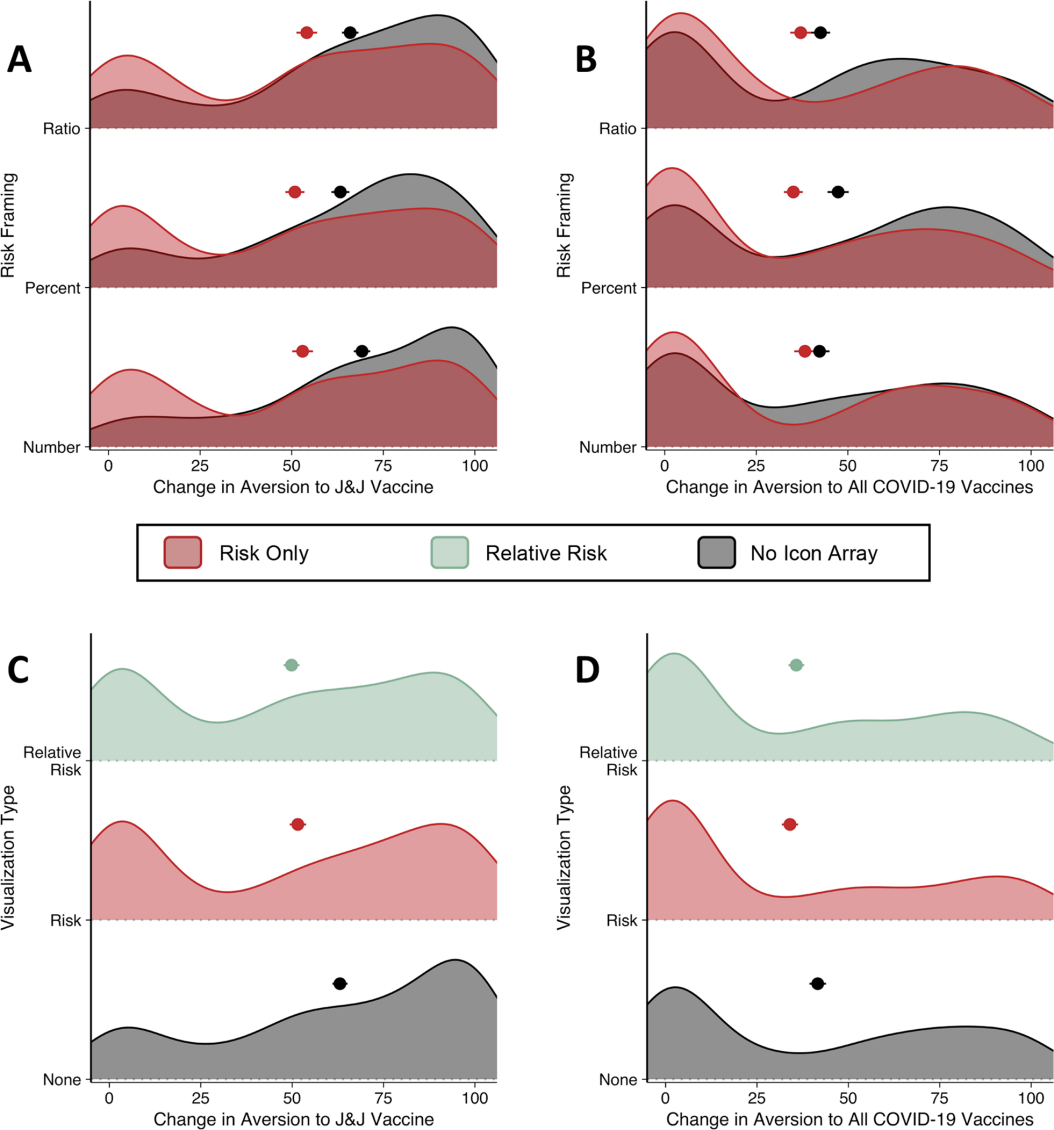
Change in Aversion toward the J&J and all COVID-19 vaccines by experiment and condition. **a**, **b** Mean and standard error change in vaccine aversion by condition in Experiment 1. Notice that the data are displayed as overlapping distributions. Point color indicates probability expression group (see legend). **c**, **d** Mean and standard error change in vaccine aversion by condition in Experiment 2. Note that while the y-axes above range from 0.25 to 0.75, the full range was 0 to 1 and that the data are displayed as stacked distributions

The two dependent variables were modeled using zero–one-inflated beta-distributional regression models, given that the data were not normally distributed and could only take on values between (and including) zero and one (see Fig. 2). The zero–one-inflated beta distribution is a mixture of a beta distribution (for intermediate values between 0 and 1) and a Bernoulli distribution (for extreme values, 0 and 1) via a mixing parameter γ ∈ [0, 1]. Intermediate scores between 0 and 1 were described using a beta distribution parameterized with mean (μ) and precision (φ). For scores equal to 0 (no change in aversion) or 1 (large increase in aversion), the probability that the response equals 1 is described by a Bernoulli distribution with a probability parameter (α).

Models for Experiment 1 included the following covariates: vaccination status (vaccinated–unvaccinated), framing condition (percent—number, ratio—number), visualization condition (icon array—none), z-scored subjective numeracy, and the interaction between framing and visualization. Regression formulae for location parameters (μ and α) included all covariates listed above; however, regression formulae for the auxiliary parameters (φ and γ) omitted numeracy and interactions between framing and visualization. We implemented the model using the R-package brms: Bayesian Regression Models using ‘Stan’ (Bürkner, 2017, 2018). Brms translates input models into the probabilistic programming language Stan, enabling approximate Bayesian inference over model parameters using Markov chain Monte Carlo (MCMC) sampling (Carpenter et al., 2017). We assigned weakly informative normal (0,1) priors to regression coefficients and used the default priors provided by brms for all other parameters (v2.14.4).

The model passed all convergence and efficiency diagnostic tests (see Vehtari et al., 2021 for more information). After fitting the models, we performed graphical posterior predictive checks using the R packages {bayesplot} (Gabry et al., 2019) and {loo} (Vehtari et al., 2017). To quantify uncertainty about the effects of interest, we computed 95% credible intervals (CI) as well as probabilities of direction (*pd*). The *pd* is defined as the probability that an effect goes in the direction indicated by the median estimate (Makowski et al., 2019). For ease of interpretation, we replicate the findings below with factorial ANOVA and report these results in Additional file 1. See Table 2 for descriptive statistics.

**Table 2:** Change in aversion toward vaccination by condition for experiments 1 and 2

Probability expression	Experiment 1							
	Change in aversion to J&J vaccine				Change in aversion to *All* COVID-19 vaccines			
	No icon array		Icon array		No icon array		Icon array	
	M(SD)	N	M(SD)	N	M(SD)	N	M(SD)	N
Number-only	.69 (.30)	179	.53 (.36)	163	.42 (.36)	179	.38 (.38)	163
Ratio	.66 (.32)	196	.54 (.36)	161	.43 (.36)	196	.37 (.36)	161
Percentage	.63 (.31)	158	.51 (.36)	195	.47 (.36)	158	.35 (.36)	195

Experiment 2											
Change in aversion to J&J vaccine						Change in aversion to *All* COVID-19 vaccines					
No icon array		Icon array (side effect)		Icon array (relative risk)		No icon array		Icon array (side effect)		Icon array (relative risk)	
M(SD)	N	M(SD)	N	M(SD)	N	M(SD)	N	M(SD)	N	M(SD)	N
.63 (.34)	278	.52 (.38)	293	.50 (.36)	280	.42 (.38)	278	.34 (.38)	293	.36 (.36)	280

### Results

First, we examine the influence of condition on increases in aversion toward the J&J vaccine. Our main finding in Experiment 1 is that participants reported lower increases in aversion toward the J&J vaccine if they viewed an icon array [M(SD) = 0.53(0.36)] compared to no visualization [M(SD) = 0.66(0.31)] (β = − 0.34, CI = [− 0.59, − 0.08], pd = 1). After viewing an icon array, participants were also more likely to report no increase in aversion (0) rather than a large increase in aversion (1) toward the J&J vaccine (β = − 0.99, CI = [− 1.89, − 0.02], pd = 0.98). In contrast to the noticeable effect of visualization, there was no evidence for effects of probability expression (all pd ≤ 0.59) nor interactions between probability expression and the presence of an icon array for intermediate values (all pd ≤ 0.76). There was some evidence that participants were more likely to report a large increase in aversion (1) than no change increase in aversion (0) toward the J&J vaccine if risk was presented as a single number rather than a ratio (β = 1.04, CI = [− 0.17, 2.26], pd = 0.96), and participants were more likely to report no increase in aversion (0) rather than a large increase in aversion (1) if risk was presented as a percentage rather than a ratio (β = − 0.7, CI = [− 1.75, 0.35], pd = 0.91) (see Fig. 2a).

Next, we examined the influence of condition on changes in aversion toward *all* COVID-19 vaccines. After viewing an icon array, participants were more likely to report no increase in aversion (0) rather than a large increase in aversion (1) toward *all* COVID-19 vaccines (β = -1.02, CI = [-2.26, 0.07], pd = 0.96). However, icon array presence did not affect increases in aversion for those reporting intermediate vaccine aversion scores between 0 and 1 (β = -0.004, CI = [-0.27, 0.26], pd = 0.51). There was little evidence for effects of probability expression (all pd ≤ 0.85) or interactions between probability expression and the presence of an icon array (all pd ≤ 0.72) (see Fig. 2b).

### Discussion

Experiment 1 found little evidence for an effect of probability expression on increases in aversion toward vaccination. There was strong evidence that viewing an icon array prevented increases in aversion toward the J&J vaccine and some evidence that such visualizations prevented increases in aversion toward *all* COVID-19 vaccines. These results suggest that viewing an icon array illustrating the potential *risks* of vaccination prevented large increases in aversion toward vaccination. In Experiment 2, we examine whether aversion could be further prevented by viewing an icon array showing both the risks and *potential benefits* of vaccination.

## Experiment 2

**Fig. 3:**
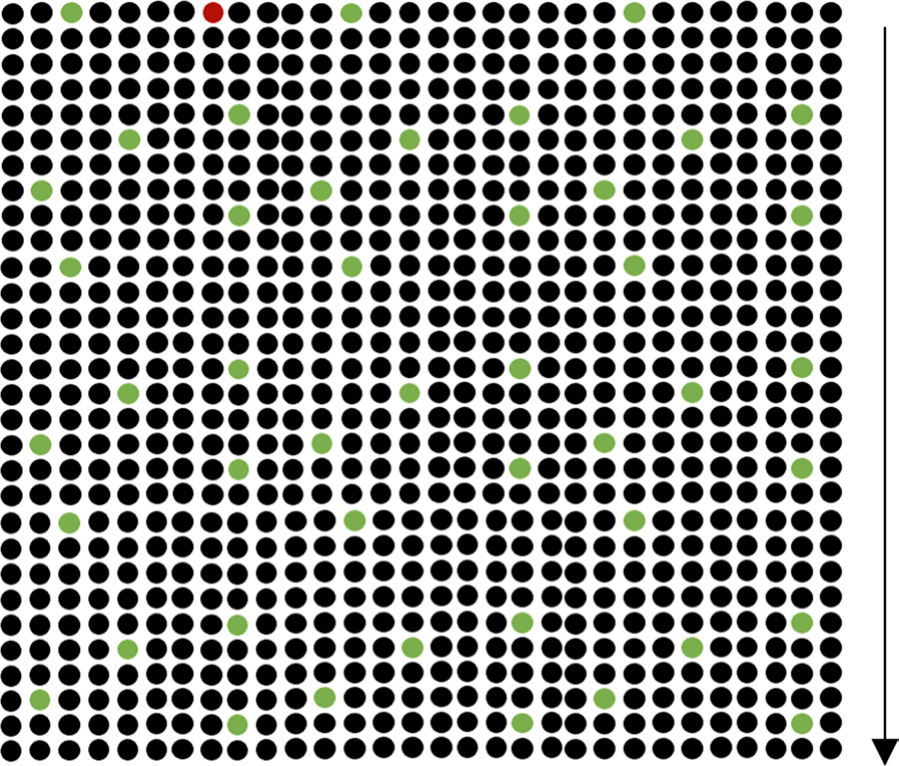
Relative risk, where 1 (red dot) in 900 experiences a side effect and 1 (green) in 20 lives is saved by the treatment. The relative-risk icon array in Experiment 2 contained 1 million dots that participants had to scroll through if assigned to a visualization condition. The arrow on the right represents how participants had to scroll through the array of dots, but this arrow was not part of the original figure

Interpretation of risks is context-dependent, so viewing the relative risk between vaccine and disease consequences may improve decision making (Reyna, 2008). Thus, in Experiment 2 we included another visualization condition showing the expected lives saved by the vaccine in addition to the risk of incurring the blood clotting side effect (1 million dots with 1 red dot representing risk of side effect and 10,000 green dots representing lives saved, assuming that 1 in 10 unvaccinated people contract COVID-19 and that 1 in 100 of those who contract COVID-19 die (Fig. 3) (Philip Bump, 2021)).

### Methods

#### Participants

Data were collected from 903 participants from Amazon MTurk. Fifty-two participants were excluded from the analyses for failing an attention check, leaving 851 participants. See demographics in Table 3.

**Table 3:** Demographic characteristics of participants in Experiment 2

Age M (SD)	Gender		Education	
38.81 (14.37)	Female	61.31%	Some High School	.11%
	Male	38.02%	High School	6.58%
	Other	.7%	Some College	13.87%
			2-years degree	8.70%
			4-years degree	47.83%
			Advanced degree	22.91%

#### Design and materials

Experiment 2 was a between-subjects design where participants were randomly assigned to view one of three visualizations: no visualization, the side-effect-only icon array from Experiment 1, or the relative-risk icon array illustrating both disease and vaccine risk (see Fig. 3). All participants viewed the probability expressed as a ratio since there was little evidence for an effect of probability expression in Experiment 1.

#### Modeling methods

Models for Experiment 2 included only vaccination status, visualization condition, and z-scored subjective numeracy as covariates.

### Results

In Experiment 2, we successfully replicated the key results of Experiment 1. Participants self-reported lower increase in aversion to the J&J vaccine if they viewed an icon array illustrating probability of side effect [M(SD) = 0.52(0.38)] compared to no visualization [M(SD) = 0.63(0.33)]. Viewing this icon array also prevented increases in aversion for those with intermediate scores (β = − 0.24, CI = [− 0.43, − 0.06], pd = 0.98). Participants were again more likely to report no increase in aversion (0) rather than a large increase in aversion (1) after viewing the icon array (β = − 1.48, CI = [− 2.05, − 0.89], pd = 1). Viewing an icon array of relative risk was also associated with lower increases in vaccine aversion when compared to the no-visualization condition [M(SD) = 0.49(0.36)] (β = − 0.20, CI = [− 0.37, − 0.02], pd = 0.96). Participants viewing the relative risk visualization were also more likely to report no increase in aversion rather than a large increase in aversion (β = − 1.75, CI = [− 2.39, − 1.13], pd = 1). The relative-risk and side-effect-only icon arrays appear to be equally effective in preventing increases in vaccine aversion (see SI; Fig. 2c).

Viewing the side-effect-only icon array was associated with lower increases in vaccine aversion for intermediate values (β = − 0.27, CI = [− 0.46, − 0.07], pd = 0.98), but the presence of an icon array did not affect the probability of reporting large increases in aversion rather than no increase in aversion (β = − 0.40, CI = [− 1.00, 0.20], pd = 0.86). Increases in vaccine aversion after viewing the relative-risk icon array were no different from viewing no visualization (β = − 0.06, CI = [− 0.25, − 0.12], pd = 0.69). After viewing the relative-risk icon array, people were more likely to report no increase in aversion, rather than a large increase in aversion (β = − 0.77, CI = [− 1.48, − 0.01], pd = 0.96) (see Fig. 2d).

### Discussion

Experiment 2 replicates the main finding from Experiment 1 that viewing icon arrays of small side-effect risk prevented increase in aversion toward the J&J vaccine. There was also some evidence that viewing these icon arrays prevented increased aversion toward *all* COVID-19 vaccines more generally. There was no evidence suggesting that viewing the relative-risk icon array was more beneficial than viewing a side-effect-only icon array.

## General discussion

The main takeaway from this research is that presenting icon arrays illustrating the very small risk of experiencing side effects in tandem with the announcement from the CDC could have minimized increases in vaccine hesitancy to both the J&J and possibly *all* COVID-19 vaccines. These results provide evidence that icon arrays are effective at communicating risk information outside of the laboratory, in a real-world context with real-world consequences. We are optimistic that our findings contribute to the literature on risk-perception more generally, as other work shows icon arrays to similarly improve decision making in many different contexts (e.g., Galesic et al., 2009; Garcia-Retamero et al., 2010; Okan et al., 2012; Walker et al, 2022; Waters et al., 2007a; Zikmund-Fisher et al., 2008), although some evidence is mixed (e.g., Recchia et al., 2022; Ruiz et al., 2013; Waters et al., 2007b). Given that much of the prior work on icon arrays has been in the context of hypothetical scenarios, while the current study was in the context of real-world decision making, we also provide evidence that icon arrays are effective in more than just hypothetical decision making.

Another contribution of our work is the finding that icon arrays can effectively communicate very small risks (0.0001%). However, it is possible that the presence of the single red dot in the array did not matter and that the visualization prevented increases in vaccine hesitancy by helping participants understand the magnitude of 1 million. Prior work shows that it is difficult for everyday people to conceptualize very large numbers, such as 1 million (see Landy et al., 2013). The icon array provides a concrete representation of an abstract idea by showing participants 1 million icons. By scrolling through the icon array, this may help participants understand just how large 1 million is. This could also explain why we find no difference between the side-effect-only and relative-risk icon arrays in Experiment 2. Alternatively, the main reason why icon arrays are thought to be beneficial in reasoning about probabilities is that they highlight the denominator (Garcia-Retamero & Cokely, 2013). If providing this concrete representation helps people better understand the magnitude of 1 million, it may also help them understand the magnitude of the denominator. Thus, it is possible that the icon array both helped participant conceptualize the magnitude of 1 million and overcome denominator neglect. Future research should disentangle these possibilities.

Conceptually, scrolling through an icon array of 1 million icons may help people understand risk magnitude through other cognitive mechanisms. Padilla et al. (2018) present a dual model of visualization processing for decision making, where type I processing is heuristic-based and open to perceptual biases, while Type II processing is more effortful and is associated with higher levels of accuracy in graph-based reasoning. Scrolling through the icon array displaying very small risk may help people engage with the visualization through a type II pathway as the visualization provides viewers with both a temporally coded and visually coded risk estimate.

One alternative explanation for the findings is that viewing the visualization made the data appear more trustworthy, resulting in lower increases in vaccine hesitancy. Some prior work has found that other types of data visualization, such as bar graphs (Tal & Wansink, 2016), increase the perceived credibility of data. However, more recent work has cast doubt on the validity of these findings (see Dragicevic & Jansen, 2017; Fansher et al., 2022). Future work could explore if including icon arrays influences the perceived trustworthiness of data.

### Limitations

One limitation of the current study is that we did not compare the effectiveness of icon arrays to other types of data visualizations. It is possible that icon arrays were more effective because they repeated the information given in the vignette graphically. However, we have reason to believe that icon arrays helped participants understand risk magnitude beyond repetition, given that other studies that have compared icon arrays to other types of data visualizations (without controlling for repetition) have found icon arrays to be most effective (e.g., Tait et al., 2010; Waters et al., 2007a, b). Another limitation is that participants self-reported their changes in attitudes toward vaccination. Ideally, we would have measured vaccine hesitancy both before and after the announcement (which, of course, was logistically not possible). One alternative explanation, and possible limitation, of the finding that there was no difference between the side-effect-only icon array and relative-risk icon array in Experiment 2, is that our participants were not tested for red/green colorblindness. To test this possibility, since colorblindness is a sex-linked trait, we reran the Experiment 2 analysis with only the females in our sample and still found no difference between groups (*p* ≥  0.42). This suggests that possible red/green colorblindness in our participants did not significantly influence our results. Lastly, it is possible that the high complexity of the language we used (i.e., “more hesitant”) introducing construct-irrelevant variance because the instructions may not have been understood equally well by all participants.

### Conclusion

Regardless of these limitations, we believe our results suggest that icon arrays can prevent large increases in vaccine hesitancy from small risks. Future work could examine whether such techniques would also be beneficial at communicating small probabilities in contexts other than side effect risk and vaccine hesitancy. For example, in the context of COVID, other potential side effect risks beyond the blood-clotting side effect could be examined. Caution should be taken when communicating information about such side effects to the public, especially given that people tend to take no action if the action is perceived to potentially cause harm, even if there is a greater risk of inaction (i.e., abstaining from vaccination (Bond & Nolan, 2011).

## Supplementary Information

**Additional file 1.** Supplemental Material.

## Data Availability

All data and materials will be deposited on OSF once paper is accepted for publication.

## Abbreviation

J&J: Johnson and Johnson
