# Supplementary material for: Icon arrays reduce concern over COVID-19 vaccine side effects: a randomized control study
Source: Cogn Res Princ Implic. 2022 May 7;7:38. doi: 10.1186/s41235-022-00387-5 (PMC9077983; doi:10.1186/s41235-022-00387-5)
Supplement: Supplementary file 1 — Additional file 1. Supplemental Material. [file 41235_2022_387_MOESM1_ESM.docx]

**Supplementary Information for**

Icon Arrays Reduce Concern Over COVID-19 Vaccine Side Effects: A Randomized Control Study

Madison Fansher, Tyler J. Adkins, Poortata Lalwani, Aysecan Boduroglu, Madison Carlson, Madelyn Quirk, Richard L. Lewis, Priti Shah, Han Zhang, John Jonides

**Corresponding Authors:** Madison Fansher, Tyler J. Adkins

**Email:** [mfansher@umich.edu](mailto:mfansher@umich.edu), [adkinsty@umich.edu](mailto:adkinsty@umich.edu)

**This PDF file includes:**

ANOVA Replication

**ANOVA Replication**

While zero-one-inflated Beta regression is arguably the most suitable approach to analyzing slider scale data, we sought to replicate our findings using a simpler analysis of variance (ANOVA). Experiment 1 data were analyzed with a factorial ANOVA with probability expression and visualization condition as factors. Data for Experiment 2 were analyzed with a one-way ANOVA with Tukey HSD post-hoc tests and visualization condition as the predictor. Significance was assessed at the .05 level and all tests were two-tailed with aversion toward the J&J vaccine or all COVID-19 vaccines as the dependent variable. Data were analyzed with the stat package in R.

In Experiment 1, probability expression did not impact aversion toward the J&J vaccine (F(2,1046) = 2.27, p = 0.1), nor all COVID-19 vaccines (F(2,1046) = .02, p = 0.98). However, participants who viewed the icon array depicting side effect risk reported significantly less aversion towards the J&J (F(1,1046) = 42.19, p < .001) and all COVID-19 vaccines (F(1,1046) = 10.31, p = .001) when compared to participants who did not view an icon array. There was no interaction between probability expression and presence of an icon array on aversion towards the J&J vaccine (F(2,1046) = 0.43, p = 0.65) nor all COVID-19 vaccines (F(2,1046) = 1.27, p = .28).

In Experiment 2 we find an overall effect of visualization condition on aversion towards the J&J (F(2, 848) = 10.9, p <.001) and all COVID-19 vaccines (F(2, 848) = 3.21, p = .04). Tukey post-hoc tests reveal that those who viewed the icon array depicting side effect risk were significantly less averse toward the J&J (M_diff_ = .12, 95%CI = [-.19, -.04], p < .001) and all COVID-19 vaccines (M_diff_ = .08, 95%CI = [-.15, -.002], p = .04) than those who viewed no icon array. Those who viewed the relative risk icon array were significantly less averse than those who viewed no visualization for the J&J vaccine (M_diff_ = .13, 95%CI = [-.20, -.06], p < 0.001) but not for all COVID-19 vaccines (M_diff_ = .06, 95%CI = [-.13, .02], p < 0.15). There was no difference in aversion toward the J&J vaccine (M_diff_ = .02, 95%CI = [-.08, .05], p = .83) or all COVID-19 vaccines (M_diff_ = .02, 95%CI = [-.06, .09], p = .84) when comparing the side effect-only and relative risk icon array conditions, suggesting the visualizations are equally effective.
